# Recombinant protein expression by targeting pre-selected chromosomal loci

**DOI:** 10.1186/1472-6750-9-100

**Published:** 2009-12-14

**Authors:** Kristina Nehlsen, Roland Schucht, Leonor da Gama-Norton, Wolfgang Krömer, Alexandra Baer, Aziz Cayli, Hansjörg Hauser, Dagmar Wirth

**Affiliations:** 1 Helmholtz Centre for Infection Research, Braunschweig, Germany; 2 Instituto de Biologia Experimental e Tecnológica, Universidade Nova de Lisboa (IBET/ITQB/UNL), Oeiras, Portugal; 3 CELLCA GmbH, Laupheim, Germany; 4 Symphogen, Kopenhagen, Denmark

## Abstract

**Background:**

Recombinant protein expression in mammalian cells is mostly achieved by stable integration of transgenes into the chromosomal DNA of established cell lines. The chromosomal surroundings have strong influences on the expression of transgenes. The exploitation of defined loci by targeting expression constructs with different regulatory elements is an approach to design high level expression systems. Further, this allows to evaluate the impact of chromosomal surroundings on distinct vector constructs.

**Results:**

We explored antibody expression upon targeting diverse expression constructs into previously tagged loci in CHO-K1 and HEK293 cells that exhibit high reporter gene expression. These loci were selected by random transfer of reporter cassettes and subsequent screening. Both, retroviral infection and plasmid transfection with eGFP or antibody expression cassettes were employed for tagging. The tagged cell clones were screened for expression and single copy integration. Cell clones producing > 20 pg/cell in 24 hours could be identified. Selected integration sites that had been flanked with heterologous recombinase target sites (FRTs) were targeted by Flp recombinase mediated cassette exchange (RMCE). The results give proof of principle for consistent protein expression upon RMCE. Upon targeting antibody expression cassettes 90-100% of all resulting cell clones showed correct integration. Antibody production was found to be highly consistent within the individual cell clones as expected from their isogenic nature. However, the nature and orientation of expression control elements revealed to be critical. The impact of different promoters was examined with the tag-and-targeting approach. For each of the chosen promoters high expression sites were identified. However, each site supported the chosen promoters to a different extent, indicating that the strength of a particular promoter is dominantly defined by its chromosomal context.

**Conclusion:**

RMCE provides a powerful method to specifically design vectors for optimized gene expression with high accuracy. Upon considering the specific requirements of chromosomal sites this method provides a unique tool to exploit such sites for predictable expression of biotechnologically relevant proteins such as antibodies.

## Background

High level expression of proteins from mammalian cells is crucial for diverse questions in basic research such as structure analysis and is a key issue for biopharmaceutical production. The current state of the art for establishment of recombinant protein production cell lines relies on transfection of producer cells with a plasmid that encodes the gene of interest driven by a potent promoter. Upon uptake into the nucleus, the incoming DNA, in particular through double-strand breaks, is sensed by the cellular repair machinery. These enzymes stably integrate the incoming recombinant DNA into the cellular DNA by illegitimate recombination. This procedure is largely random and accordingly, the sites of integration are mostly spread all over the genome [1].

Once integrated into the cellular DNA, the transgene cassette is affected by neighboring chromosomal elements that modulate the promoter to a high extent (see West and Fraser, [2] for a recent review). Enhancers and silencers directly affect promoters in *cis *and may be shielded by insulators. Beside this, chromatin modeling elements such as locus control regions and S/MARs significantly influence the transgene expression level [2-4]. Finally, evidence has been provided that also nearby/close promoter elements interact with incoming promoters (promoter crosstalk) and can result in their downregulation (so-called promoter occlusion) or potentiation [5]. Thus, upon random integration, individual cell clones display a highly heterogenous expression pattern and have to be screened for appropriate expression.

Homologous recombination is used in stem cells for targeting transgene to specific loci. In differentiated cells homologous recombination is very infrequent. Recently, Zn-finger nuclease based approaches have been designed for targeting transgene cassettes in mammalian cells to defined loci (reviewed in [6,7]). Thus, tools are available to target individual chromosomal sites for various applications in order to overcome the limitations of random integrations. While such methods are useful for gene therapies and basic research, their value for protein expression is limited since chromosomal sites in production cell lines that support high level recombinant protein expression are usually not known.

Indeed, in order to meet the requirements for high and stable protein expression extensive screenings are performed to identify those cell lines that provide optimal protein production. For industrial purposes devices for robotic cell and sample propagation were developed that support high throughput screenings of millions of individual cell clones, thereby allowing the identification of those cell clones with favorable expression of the transgene. Beside this, procedures for enhancing the copy number of transgene integrations by gene amplification have been employed (see e.g. [8,9]). However, high clone to clone variations as well as instability in expression levels have been found. The latter is due to genetic rearrangements during gene amplification as well as to the emergence of drug-resistance [10-12]. Although these procedures have provided potent producer clones, the pitfalls are obvious: for any and every new protein and/or expression construct the screening process has to be re-established which is both time consuming and expensive. Alternative methods to decrease the production cost and the time needed for cell line development are thus essential.

In the last two decades specific genetic engineering of mammalian cells via site specific recombinases such as Cre and Flp has been explored. These recombinases specifically bind and recombine short recombination target sites, the 34 bp loxP site and 48 bp FRT site, respectively. Once a chromosomal site in the host genome is tagged with a single recombination target sequence, the recombinases mediate site specific targeting of plasmids carrying the same recombination target site. Thereby, targeted integration of whole plasmids into pre-tagged integration sites in mammalian cells is feasible [12,13]. This first generation gene targeting strategy suffers from low efficiency due to possible excision of the integrated cassette and also from integration of extended plasmid backbone sequences. Accordingly, only few cell lines have been provided so far that allow for successful targeted integration of a transgene cassette. Although some tagged cell lines are commercially available, they are not screened for high production and do not meet the requirements for many applications.

The method of recombinase mediated targeting was significantly improved by flanking an initial tagging cassette with a set of non-interacting recombinase recognition sites. Upon integration, such cassettes can be precisely exchanged for an incoming vector that is flanked with the same set of recombinase recognition sites [13-15]. Hence, the term recombinase mediated cassette exchange (RMCE) was coined [16]. Basically, RMCE relies on two heterologous recombinase target sites (spacer mutants) that resist site specific recombination between each other but still undergo recombination with their respective homologous counterparts. Mutants that can be exploited in this respect have been identified both for the Flp [14] and the Cre system (reviewed in [17]). The main advantage of RMCE is the lack of excision which reduces the targeting efficiency in simple first generation targeted integration approaches. Upon implementation of stringent selection strategies the frequency of targeting can be increased [18] even up to 100% [19,20]. Further, RMCE overcomes the integration of bacterial vector sequences which potentially limits mammalian gene expression. Meanwhile, this technology has been exploited in various cell systems and for various applications including the establishment of viral producer cells [20,21], EPO production [22], and for the evaluation of vector design [19] (see [17] for a recent review).

A systematic exploitation of defined chromosomal sites for expression of proteins has not yet been followed. The present study focuses on the evaluation of different approaches for tagging and the evaluation of the flexibility of incoming cassettes concerning the predictability of expression upon targeting. Moreover, we give evidence that the strength of a given promoter is strongly linked to the properties of the respective chromosomal integration site. Together, the study gives new insights in the interactions between promoters and chromosomal elements. Thereby, it contributes to a deeper understanding of these mechanisms which is a prerequisite for a systematic exploitation of chromosomal integration sites for various applications including protein production.

## Methods

### Plasmids

The retroviral tagging cassette RV-GFP has been described elsewhere ('pTAGeGFP' in [20]). It contains in its 3'LTR a wild-type FRT site and a F5 spacer mutant FRT site, followed by an ATG-deleted neomycin phosphotransferase gene.

Plasmidic tagging cassettes P-HTG and P-GFP harbor the respective reporter gene(s) flanked by a wild-type FRT site and F5 spacer mutant FRT site, and are also followed by an ATG-deficient neomycin phosphotransferase gene. In P-GFP eGFP as fluorescence marker is expressed from the SV40 promoter, in P-HTG the PGK promoter drives the HTG fusion protein comprising hygromycin phosphotransferase, thymidine kinase and eGFP. The antibody coding tagging cassette harbors the SV40 driven HTG fusion and the heavy and the light chain of an IgG molecule each controlled independently by an SV40 promoter/enhancer.

Targeting cassettes contain the FRT wild-type and the F5 FRT mutant site flanking an antibody expression unit. The design of the cassettes is depicted in the individual figures. The cassettes carry the SV40 promoter, the CMV promoter, a hybrid promoter comprising the MPSV enhancer elements and the CMV promoter [23] or a bidirectional promoter composed of the Adenovirus major late gene promoter and the elongation factor 1 promoter [24]. Targeting vectors encode RFP, eGFP or antibody expression cassettes. In all targeting vectors, the PGK promoter or an IRES element and an ATG start codon is positioned upstream of the FRT mutant site to complement the inactive neoR gene after targeting. Maps or sequences are available upon request.

### Mammalian Cell Culture and Transfection

CHO-K1 cells (ATCC CCL 61) were cultivated at 37°C in a humidified atmosphere with 7.5% CO_2 _in CD Hybridoma medium (Gibco) with 2% fetal calf serum (Biowest), 8 mM L-glutamine and 4 ml of 250× Cholesterol lipid concentrate (Gibco) per litre medium. Selection was performed in medium supplemented with hygromycin B (150 U/ml), G418 (500 μg/ml) or ganciclovir (10 μg/ml). HEK293 cells (BioReliance) were cultivated at 37°C in a humidified atmosphere with 5% CO_2 _in DMEM (Gibco) with 10% fetal calf serum (Cytogen), 2 mM L-glutamine, penicillin (10 U/ml) and streptomycin sulfate (100 μg/ml). Selection was performed in medium supplemented with hygromycin B (200 U/ml), G418 (1500 μg/ml) or ganciclovir (10 μg/ml).

For plasmidic transfer CHO-K1 cells were transfected with 4 μg of the tagging vector using the nucleofection standard protocol (amaxa AG; Cologne; Germany; Nucleofector™ Kit V) and selected with hygromycin B for optimal generation of single copy clones. HEK293 cells were plasmid transfected using the GenePulser electroporator (BioRad). For this purpose 1 × 10^6 ^cells were transfected with 2.6 μg of the tagging vector carrying an eGFP cassette. eGFP positive cells were sorted using flow cytometry and individual clones were expanded. Retroviral tagging was performed as described earlier [19]. In brief, tagging viral vectors were generated upon transfection of PG13 packaging cells. The supernatant was used to infect HEK293 and CHO-K1 cells at an m.o.i. of 0.1 and the cells were subjected to selection with hygromycin B.

### Targeted Cassette Exchange

For site-specific cassette exchange 4 × 10^5 ^of tagged HEK293 cells were co-transfected with 2 μg Flp recombinase-expressing vector (pFlpe [20]) and 2 μg of targeting plasmid using lipofection (GenePORTER™ 2 Transfection Reagent, Peqlab). Targeting of tagged CHO-K1 cells was performed using nucleofection (amaxa) by cotransfer of 4 μg Flp recombinase-expressing vector and 1 μg of the targeting plasmid. For both, the medium was replaced 24 h post transfection and the cells were cultivated for 4 days to allow cassette exchange. On the fifth day the cells were transferred to a 60-mm culture plate and G418- and ganciclovir-containing medium to select for targeted daughter clones.

### PCR Analysis

Neo-resistant clones were checked for correct integration of the targeting vector by PCR. The use of a set of primers where the 5' primer is located in the newly integrated targeting cassette (e.g. the IRES element or PGK promoter) and the 3' primer located in the tagging backbone (e.g. the neomycin phosphotransferase gene) leads to the amplification of a targeting-specific product. The PCR is performed using the Mango-Taq Polymerase Kit (Bioline). The annealing temperature of the used primers (e.g. primer pair: PGKfwd 5' TCTCGCACATTCTTCACGTCC 3' and Neorev2 5' GTCATAGCCGAATAGCCTCTCC-3') was 58°C with an elongation time of 30 sec.

### Flow Cytometry

FACSCalibur and FACSVantage SE (Becton Dickinson) were used for evaluation and isolation of eGFP positive cells. The cells were washed, trypsinized and stained with propidium iodide (50 μg/ml) to exclude dead cells from the analysis.

### ELISA

The specific productivity of the clones was analysed by sandwich enzyme linked immunosorbent assay (ELISA). The cells were seeded on a 6-well plate with a density of 5 × 10^5 ^cells and incubated for 24 h with 2 ml of medium. The next day the cell number was determined, the supernatant harvested and centrifuged (5 min for 1000 rpm) and added to a 96-well plate covered with an Fc-specific anti-human IgG (SIGMA). The photometric measurement was done based on a substrate conversion by peroxidase (HRPO) labelled goat anti human IgG (H+L), (CALTAG™ Laboratories). Levels significantly above background and below 0.1 pg per cell in 24 h (pcd) are indicated as 0.01-0.1.

## Results

### Strategies for generation of targetable cell clones

**Figure 1:**
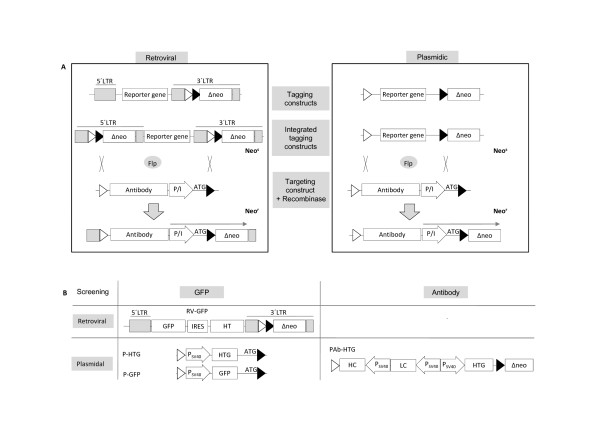
**Strategy for the identification of single copy tagged high expressing cell clones for cassette exchange**. (A) High expression chromosomal loci were tagged with either retrovirally or plasmid-mediated transduced expression cassettes. These cassettes were flanked by a set of heterospecific recombinase target sites. Tagged cells lines were analyzed for the stable integration of one single copy of the respective tagging vector and screened for high expressing integration loci. The transfer of a targeting vector carrying the same heterospecific recombinase target sites as the tagging vector in presence of the Flp-recombinase leads to a site-specific cassette exchange. The selection for successfully targeted clones was performed by complementing a silent, ATG-defective neomycin resistance gene pre-integrated upon tagging. This renders successfully targeted cells resistant to G418. For this purpose, the incoming targeting vector carried next to its gene of interest (antibody expression unit) a specific sequence (P/I) that facilitates expression of the neomycin resistance gene. (B) The vectors used for tagging are depicted. All tagging cassettes contain a promoter or internal ribosomal entry site for activation of the neomycin resistance gene and are flanked by heterospecific (Fwt-F5) FRT sites. Retroviral tagging was performed as described in [20] with a vector that transduces a bicistronic cassette of eGFP and a hygromycin phosphotransferase/thymidine kinase fusion protein. For plasmidic tagging vectors with different reporter genes (eGFP and/or antibody expression unit) as well as varying promoter elements (SV40/PGK) were employed. All tagging vectors express eGFP, either as a fusion protein with the hygromycin phosphotransferase/thymidine kinase or as a single protein, allowing fluorescence-based screening for the expression of the tagging cassette.

To generate cell clones which are suitable for targeted integration we followed a Flp recombinase based strategy as outlined in Figure 1A. It comprises the tagging of chromosomal integration sites within the host genome of a given cell line with a reporter gene cassette. Catalyzed by the Flp recombinase that is encoded by a plasmid and is co-transferred together with the targeting vector cassette exchange will occur (Flp recombinase mediated cassette exchange, RMCE, Figure 1A).

To generate high producer cell clones, different approaches for tagging chromosomal loci were evaluated (Figure 1A). We employed plasmid transduction, in particular a classical electroporation protocol for HEK293 cells and nucleofection which has been the method of choice for many cell lines including CHO [25]. Further, retroviral transduction was followed for both cell lines for two reasons: first, this methodallows to statistically adjust the copy number by using a low, defined ratio of recombinant virus particles to the number of infected cells (multiplicity of infection). Second, this method has been reported to favour high expression integration sites [26,27]. As recipients we used HEK293 and CHO-K1, both cell lines used in basic research and industrial biotechnology.

All tagging vectors include an expression unit encoding eGFP, either as a fusion protein with the hygromycin phosphotransferase/thymidine kinase (HTG) or as a single protein unit (GFP), allowing a fluorescence-based screening for the expression of the tagging cassette (see Figure 1B). Identification of high expression cell clones (screening) was either carried out by multiple rounds of FACS sorting or limited dilution cloning steps.

### Expression strength, stability and copy number of tagged cell clones

**Figure 2:**
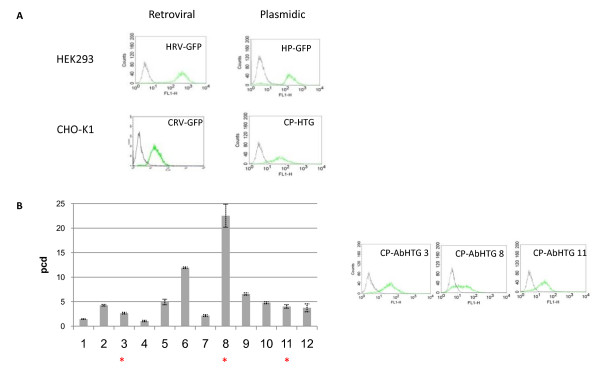
**Characterization of the tagged integration sites**. (A) GFP expression pattern of single copy HEK293 and CHOK1 clones obtained upon retroviral and plasmidic transfer of the tagging cassette. Representative clones are shown. Green lines: tagged clones. Grey lines represent non-transfected HEK293 and CHOK1 cells, respectively. (B) CHO cells were tagged with an antibody expression construct. The antibody expression level production was evaluated. Only cell clones that produced >2 pcd are shown; the data were obtained from 3-5 replicates. Clones showing single copy integration events (data not shown) are marked by asterisks. Their GFP expression profile is given presented on the right. Green lines: tagged clones; grey lines: non-transfected CHOK1 cells. Nomenclature: HRV-GFP: HEK293 cells retrovirally tagged with eGFP (RV-GFP according to figure 1B); HP-GFP: HEK293 cells plasmid-tagged with eGFP (P-GFP); CRV-GFP: CHO cells retrovirally tagged with eGFP (RV-GFP); CP-AbHTG: CHO cells plasmid- tagged with antibody cassette (PAb-HTG).

We comparatively evaluated the different methods for their capability to identify high expression cell clones from both cell lines. CHO-K1 and HEK293 cells were tagged with an eGFP reporter cassette according to Figure 1. GFP expression of representative CHO-K1 and HEK293 master cell clones for each gene transfer method is depicted in Figure 2A. In all these cell clones, eGFP expression was found to be stable for more than 6 months. In an independent approach, CHO-K1 cells were tagged with an antibody expressing construct. Cells tagged with antibody expression constructs were cloned into 96 well plates after selection of hygromycin resistance and then tested for antibody expression by ELISA and for GFP by FACS. After intensive screening of > 800 hygromycin-resistant clones, about twenty clones were identified producing more than 2 pg per cell in 24 h (pcd), which was considered an arbitrary threshold for high expression. High expressing cell clones were also analysed for their expression stability over time. This revealed that the highest expressing clones #6 and #8, producing 12.0 and 22.5 pcd, respectively, showed long term instability. Although not being considered for RMCE, clone #8 gives evidence that high level expression can be obtained from single copy integrations.

The eGFP and antibody expressing cell clones were analyzed by Southern Blot for the copy numbers of the respective tagging construct (additional file 1). This proved single copy integrations in clones #3, #8 and #11.

We further evaluated the frequency of single copy integrations with the various tagging methods. In agreement with previous studies classical electroporation protocols [28], but also nucleofection provided high efficiencies of single copy integration events (Table 1). The single copy integration rates obtained were about 30% for CHO and 56% for HEK293 cells after plasmidic transfer. As expected, a very high frequency (> 90%) of single copy integration events was achieved for retrovirally tagged cell lines. Thus, all chosen transduction protocols turned out to be suitable for efficient tagging according to the RMCE strategy. Successfully tagged single copy cell clones from the various strategies providing long term expression stability are called "master cell clones" henceforth. They were used for further evaluation of the targeting accuracy as well as the homogeneity of expression upon targeting.

**Table 1:** Single-copy integration rate of tagged high-expressing cells

	Retroviral		Plasmidal (transfer method)	
**HEK293**	44/48	92%	5/9 (EP Gene Pulser, BioRad)	56%

**CHO-K1**	2/2	100%	6/20 (Nucleofection, Amaxa)	30%

### Targeting efficiency and specificity

**Figure 3:**
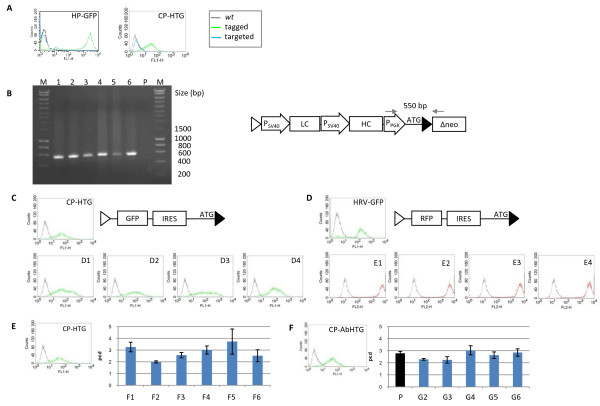
**Gene expression of cell clones after targeting by cassette exchange**. Characterization of HP-GFP and CP-GFP upon targeting. The cell clones show homogeneous loss of GFP expression after targeting with an antibody expression vector as depicted in (B). (B) Molecular characterization of the targeting event by PCR. Amplification of a 550 bp fragment from the daughter cell clones (D1-D6) using primers located at the indicated positions (arrows) is specific for integration of the targeting construct (P: parental tagged cell clone). (C) Homogeneity of the targeted daughter clones. The expression level of daughter clones after cassette exchange is compared with the expression level of the parental clones. Upper left: CP-HTG tagged clone targeted with a SV40 promoter driven GFP cassette. Upper right: HRV-GFP tagged clone targeted with a CMV-RFP cassette. Antibody targeting: CP-HTG tagged clone (lower left) and CP-AbHTG tagged clone (lower right) were targeted with an antibody expression construct as depicted in (B). The expression of the targeted daughter clones is shown.

We evaluated the efficiency and accuracy of RMCE upon targeting various eGFP and antibody expressing vectors specified in more detail below. All vectors carried an IRES element or a promoter as illustrated in Figure 1A and were co-transfected into the tagged master cell lines that had proven to stably express their reporter gene from a single copy locus. The selection of successfully targeted cells was accomplished as shown in Figure 1A. The efficiency of integrating targeting vectors into the preselected chromosomal sites was evaluated. The complementation of the defective neomycin resistance gene resulting in G418 resistant cell clones (Figure 1) was taken as a measure for the targeting efficiency. Resistant daughter cell clones were expanded for further analysis. Successfully targeted master cell clones should have lost the parental construct and show correct site-specific integration of the targeting cassette. Figure 3 demonstrates the loss of eGFP expression after exchange with an antibody encoding cassette in representative HEK293 and CHO-K1 daughter cell clones. Specific integration into the previously tagged locus was confirmed by PCR analysis as exemplified in Figure 3B. We also proved the specifically targeted cell clones for absence of additional random integration events of the targeting construct by PCR or Southern Blot analysis (see additional file 1). In Table 2, the overall targeting efficiency is given as the percentage of correctly targeted within the obtained G418-resistant clones. All HEK293 G418-resistant derived clones (daughter clones) analysed showed the specific integration of the targeting construct and lack unspecific random integration. In CHO-K1 cells the targeting efficiency was slightly lower but still above 85%. In some of the successfully targeted CHO-K1 cell clones we could find additional randomly integrated copies of the targeting construct.

**Table 2:** Targeting efficiency and additional random integration

	Targeting		Random Integration	
**HRV-GFP**	30/30*	100%	0/30	*<3%*

**CP-HTG**	48/56*	85,7%	8/56	14,3%

*targeted/neo^r ^clones

### Homogeneity of expression strength from the targeted cell clones

To evaluate the homogeneity of expression level of the daughter cell clones, three master cell clones were targeted with expression vectors encoding eGFP, RFP or heavy and light chains of an antibody. Plasmid-tagged as well as retrovirally tagged clones were chosen and individual daughter clones were characterized for expression. As illustrated in Figure 3, CHO-K1 based CP-HTG cells plasmid-tagged with the eGFP-fusion reporter construct were targeted with an eGFP (Figure 3C) and an antibody targeting vector (Figure 3D), respectively. From the analyzed targeted daughter clones, all showed a pronounced homogeneity in expression of both reporters which would be expected from isogenic clones. Accordingly, retrovirally tagged HEK293 cells (HRV-GFP) were targeted using an RFP coding vector (Figure 3E) and the RFP expression in the isogenic daughter clones again proved to be homogenous. Finally, an antibody expressing master cell line (CP-AbHTG) was targeted with an antibody cassette harbouring an expression unit with the same antibody. Again, a uniform expression pattern within the daughter clones (lower right). This setting also allowed to compare the antibody titer of the master cell clone and upon targeting. Notably, the antibody expression within the daughter clones (blue) was consistent with that of the parental cell line (black).

### Evaluation of cassette design for antibody expression

To assess the capacity of the tagged integration sites of the individual master clones to support different cassette designs and promoters, we designed a set of antibody targeting vectors differing in the architecture. In these targeting vectors, the heavy and the light chain genes are either individually transcribed from SV40 promoter elements or they are encoded in a bicistronic expression unit driven by SV40 or MPSV/CMV [23] promoter elements and employing the NRF [29] or the Poliovirus IRES element [29,30]. In addition, tricistronic expression constructs were created in which a second IRES element combines the antibody cassette with the ATG start codon that complements the defective neomycin resistance gene in the tagging locus upon targeting.

**Figure 4:**
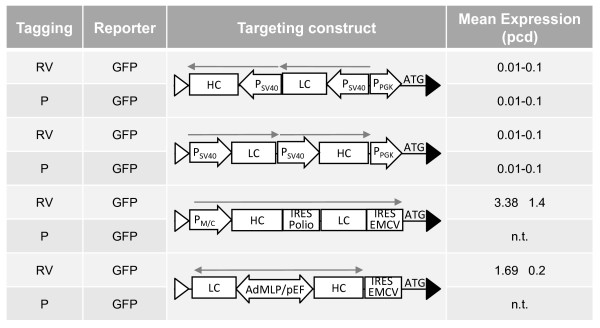
**Antibody expression after targeting in plasmid-mediated and retrovirally tagged HEK293**. The retrovirally (RV) or plasmid-mediated (P) tagged GFP expressing HEK293 cells were targeted with the indicated antibody expression cassettes. Antibody expression levels are given as mean expression of 5 targeted daughter clones (pcd). n.t.: not tested.

The mean antibody expression level of the daughter clones after targeting the indicated vectors in plasmid-tagged (P) or retrovirally (RV) tagged HEK293 master cells is shown in Figure 4. Interestingly, in these cells the targeting constructs with antibody chains individually controlled by SV40 promoter elements resulted only in basal expression levels (0.1-0.1 pcd). This was observed upon targeting in either of the two transcriptional orientations. However, upon targeting with the MPSV/CMV driven tricistronic expression cassette the retrovirally tagged clone yielded 3.4 pcd in the same locus.

**Figure 5:**
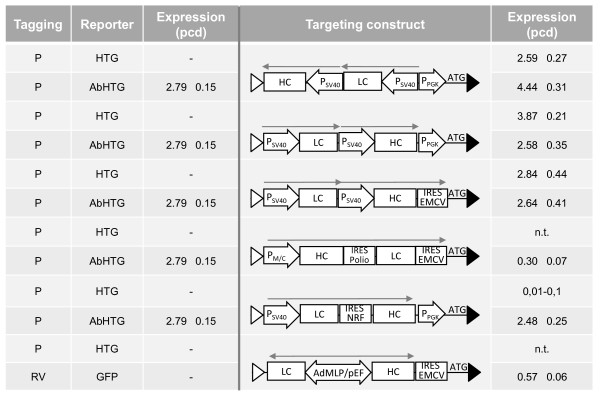
**Antibody expression after targeting of plasmid-mediated tagged CHO-K1**. CHO-K1 derived clones plasmid-mediated tagged with either a HTG or AbHTG cassette as indicated were targeted with different antibody expression cassettes. The mean expression of 5 daughter clones after targeting with the depicted constructs as well as the antibody expression of the parental cell in pcd is presented. n.t: not tested.

We evaluated the same set of antibody expression cassettes in two different plasmid-tagged loci in CHO-K1 cells, clones CP-HTG and CP-AbHTG. Figure 5 depicts the mean antibody expression level of the daughter clones. In opposite to the tagged HEK293 cells discussed before, all antibody vectors harbouring two SV40 promoters showed high expression levels (2.6 - 4.4 pcd). In contrast, in these integration sites the MPSV/CMV driven expression setup performed poorly (0.3 pcd). Interestingly, a targeting construct that harbors an SV40 driven bicistronic antibody expression cassette performed well in CP-AbHTG but failed in the CP-HTG. Together, these data indicate that specific regulatory elements are strongly modulated by flanking chromosomal elements and thus their performance is critically dependent on the nature of the specific chromosomal site.

## Discussion

High level recombinant protein expression in mammalian cells not only relies on potent transcription promoting elements and optimal design of the expression cassette but also crucially depends on appropriate chromosomal sites that support the incoming expression cassette upon integration. Sophisticated chromosomal engineering approaches based on site specific recombinases allow the precise/controlled integration of expression cassettes into tagged chromosomal sites (reviewed in [17,31,32]). A first-generation targeting system with a single recombinase target site has recently been evaluated for protein production. Comparable (consistent) antibody expression levels could be achieved upon targeting at defined integration sites in CHO cells using Cre-[33] or Flp-mediated integration [34]. The potential of this technology was further exploited for the production of a human polyclonal anti-RhD antibody [24] by integrating 25 individual antibody expression cassettes into a defined FRT tagged integration site in CHO cells (Flp-In™ cell line; Invitrogen). Irrespective of this success first generation targeting systems are limited by fact that the tagging sequences cannot be eliminated. Usually, complete vectors including bacterial sequences are co-integrated which have been shown to decrease transgene expression from neighboring promoters (e.g. [35,36]). Also, since excision of the targeted cassette is favored, the targeting efficiency can be unsatisfactory. In this respect, exchange of tagging cassettes via RMCE seems to be the method of choice. While proof of principle has been given for production of retroviral vectors [20,21] so far, a systematic approach evaluating RMCE for protein production has been missing.

This report describes a comparative approach to identify potent integration sites that support stable protein production and to exploit these sites by Flp RMCE to target expression cassettes of choice. For this purpose we used CHO-K1 and HEK293 cells - the most relevant cell lines for protein production. Motivated by the notion that any tagging procedure has an intrinsic bias for specific patterns/types of integration sites we employed random tagging strategies based on retroviral or plasmidic transfer of screening vectors. For monitoring expression we employed eGFP (either in a single expression unit or in a fusion to a selection marker) or an IgG molecule. With these screening approaches we established a set of tagged master cell lines that stably express the respective reporter gene(s). Importantly, both plasmidic and retroviral tagging proved to be appropriate for RMCE since they lead to a high-percentage of single copy integrants ranging from 30 to > 90% - a prerequisite for targeted integration.

Evaluation of the performance of cassette exchange in the different master cell clones derived from HEK293 and CHO-K1 cells using various targeting vectors proved to be highly efficient with ≥85% correctly targeted daughter clones. In certain applications, this high efficiency might overcome the need for subsequent sub-cloning. Further, analysis of the production levels could confirm that site directed integration significantly reduces the variations of clonal expression levels. This is expected from isogenic clones and is in accordance to previous reports evaluating this method for production of retroviral vectors [20].

Further, we investigated the flexibility of the tagged integration sites with respect to supporting other promoters. Unexpectedly, in the master clones HP-GFP and HRV-GFP targeting of SV40 based antibody cassettes failed to provide significant levels of antibody expression (Figure 4). In contrast, high level antibody expression was obtained in these integration sites upon targeting either an MPSV/CMV chimeric promoter or a bidirectional composite promoter, resulting in 3.38 and 1.69 pcd, respectively. Interestingly, the opposite situation was observed in CHO-K1 cell clones: while targeting of the SV40 promoter cassettes resulted in high level antibody expression in the range of 2.5-4.4 pcd, performance of promoters such as the composite MPSV/CMV and bidirectional AdMLP/pEF promoters was significantly impaired (Figure 5).

Several reports and also results from our lab (e.g. [37]; data not shown) give evidence that the SV40 promoter is a potent, although not the most favourable promoter in both CHO and HEK293 cells. This rules out that this differential performance obtained upon targeting is a consequence of a diverse set of transcription factors differentially supporting in these two cell lines. Rather, it seems to be that the nature/composition of the initial tagging vector would define the capacity of the integration site with respect to supporting promoters: the master cell clones HP-GFP and HRV-GFP which were incompatible with SV40 based targeting vectors were tagged and screened for high level GFP expression from a PGK promoter and an MSCV promoter, respectively. In contrast, the master cell clones CP-HTG and CP-ABHTG which showed high level of SV40 based expression upon targeting were initially screened to support an SV40 promoter driven tagging vector. Together, these data indicate that specific promoters show preferential performances in certain integration sites.

Our data from the screening for high expression clones show that chromosomal sites that support high level expression can be identified with both promoters. However, it seems that the nature of the integration site specifically defines the final strength of a given promoter. This interpretation is not immediately compatible with the general believe that certain promoters are particularly strong in certain cell lines. It is important to note that the data that led to this conclusion are derived from transient expression experiments or from experiments in which pools of transfectants were analyzed. For transient expression the composition of soluble (transcription) factors might indeed constitute the dominant level of promoter strength [38]. However, upon stable integration into the host genome the influence of the surrounding chromatin might be dominant over the influence of the soluble factors, given that the composition of the promoter allows expression at all.

This suggests that the strength/potential of a specific integration site is linked to a certain promoter - and is not necessarily supporting any integrated expression cassette. For application of the tag-and targeting approach it indicates that the molecular composition of targeting vectors and chromosomal integration site go hand-in-hand. Thus, it will be important to consider the specific requirements of a particular integration site relating to the maximum level of recombinant protein production that can be achieved.

Various types of chromosomal elements have been identified that contribute and modulate individual expression cassettes upon integration [2-4,39]. In the last years increasing evidence has been provided showing that not only specific genetic elements but also complex epigenetic mechanisms can be involved. We employed RMCE to test if expression from a weak integration site can be increased by chromosomal engineering of the integration site. However, neither the cHS4 element nor a potent S/MAR could significantly (more than 2 fold) increase the level of expression (data not shown). This indicates that the mere integration of chromosomal elements into specific loci is not of benefit *per se *but would require certain prerequisites. At the same time this gives evidence that our knowledge about the chromosomal elements and their influence on transgene expression is still rudimental. This might explain why the rational construction of synthetic expression domains providing *per se *all the needs for position independent and high expression is not straight forward and is still in its infancy. With the technologies now available for targeting transgenes to pre-defined loci our understanding of mechanism governing the crosstalk of chromosomal elements should be broadened.

## Conclusions

RMCE provides a powerful strategy to specifically adapt vector designs for optimized gene expression to the specific requirements of chromosomal sites. Thereby, this method provides a unique tool to exploit such sites for predictable expression of biotechnologically relevant proteins such as antibodies.

## Authors' contributions

KN, RS, HH and DW conceived and designed the experiments. KN, RS and LGN performed the experiments and analyzed the data. LGN, AB, AC and WK helped drafting the manuscript. KN, DW and HH wrote the manuscript. All authors edited, read and approved the final manuscript.

## Supplementary Material

Additional file 1 **Evaluation of the number of integrated copies**. A Southern blot analysis was performed for P-HTG tagged HEK293 cells and P-Ab-HTG antibody tagged CHO cells to detect the number of bordering fragments. Click here for file
