# Supplementary material for: Recombinant protein expression by targeting pre-selected chromosomal loci
Source: BMC Biotechnol. 2009 Dec 14;9:100. doi: 10.1186/1472-6750-9-100 (PMC2804664; doi:10.1186/1472-6750-9-100)
Supplement: Additional file 1 — Evaluation of the number of integrated copies. A Southern blot analysis was performed for P-HTG tagged HEK293 cells and P-Ab-HTG antibody tagged CHO cells to detect the number of bordering fragments. [file 1472-6750-9-100-S1.PPT]

## Slide 1
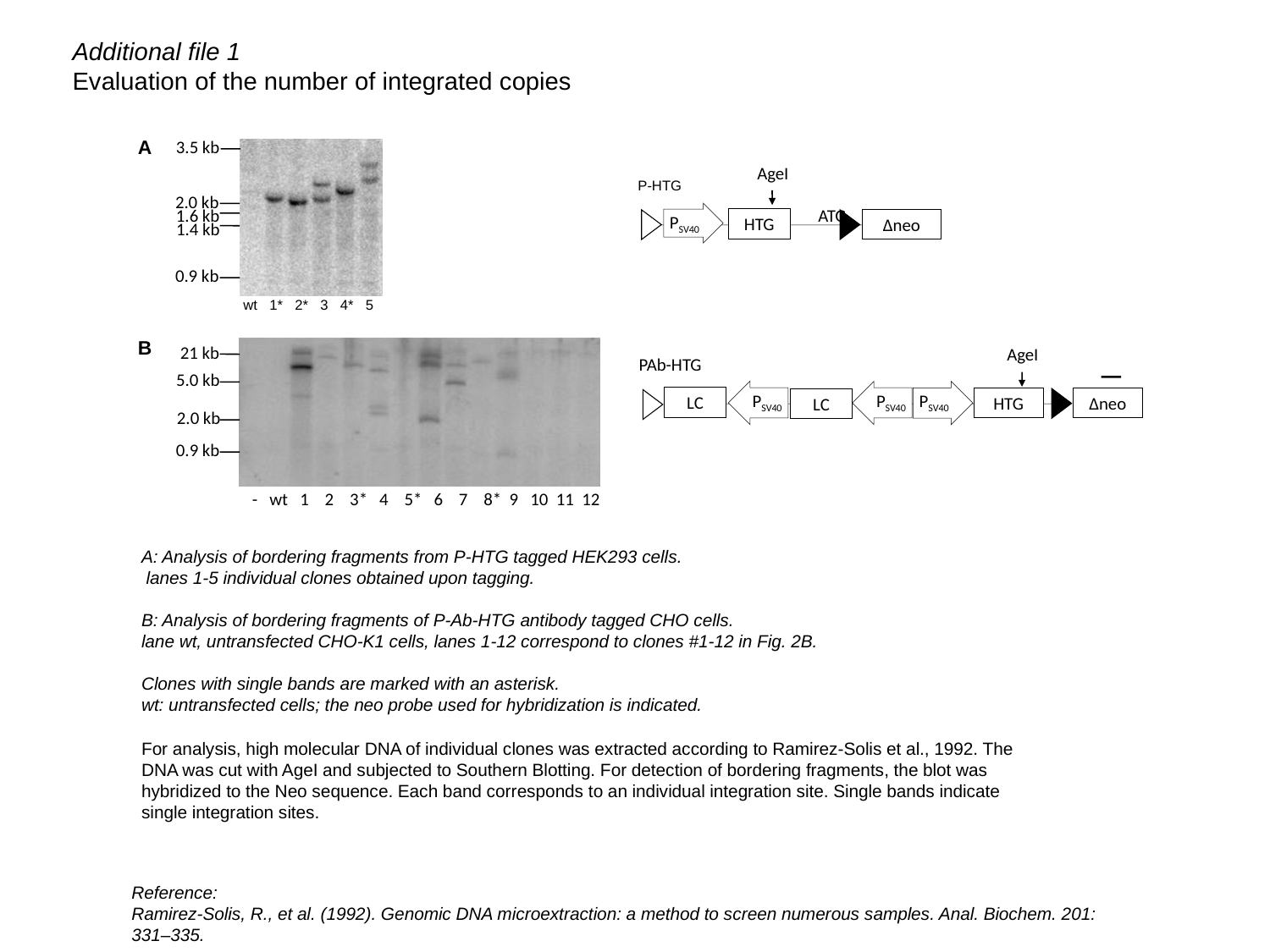

Additional file 1
Evaluation of the number of integrated copies
A
3.5 kb
AgeI
P-HTG
2.0 kb
1.6 kb
ATG
PSV40
HTG
∆neo
1.4 kb
0.9 kb
 wt 1* 2* 3 4* 5
B
21 kb
AgeI
PAb-HTG
PSV40
PSV40
PSV40
LC
HTG
∆neo
LC
5.0 kb
2.0 kb
0.9 kb
 - wt 1 2 3* 4 5* 6 7 8* 9 10 11 12
A: Analysis of bordering fragments from P-HTG tagged HEK293 cells.
 lanes 1-5 individual clones obtained upon tagging.
B: Analysis of bordering fragments of P-Ab-HTG antibody tagged CHO cells.
lane wt, untransfected CHO-K1 cells, lanes 1-12 correspond to clones #1-12 in Fig. 2B.
Clones with single bands are marked with an asterisk.
wt: untransfected cells; the neo probe used for hybridization is indicated.
For analysis, high molecular DNA of individual clones was extracted according to Ramirez-Solis et al., 1992. The DNA was cut with AgeI and subjected to Southern Blotting. For detection of bordering fragments, the blot was hybridized to the Neo sequence. Each band corresponds to an individual integration site. Single bands indicate single integration sites.
Reference:
Ramirez-Solis, R., et al. (1992). Genomic DNA microextraction: a method to screen numerous samples. Anal. Biochem. 201: 331–335.
